# Molecular Characterization and Functional Analysis of a Novel Calcium-Dependent Protein Kinase 4 from *Eimeria tenella*

**DOI:** 10.1371/journal.pone.0168132

**Published:** 2016-12-15

**Authors:** Ziwen Wang, Bing Huang, Hui Dong, Qiping Zhao, Shunhai Zhu, Weili Xia, Shuaibin Xu, Yuxiang Xie, Xiaoxia Cui, Min Tang, Qifei Men, Zhiyuang Yang, Cong Li, Xuelong Zhu, Hongyu Han

**Affiliations:** 1 Shanghai Veterinary Research Institute, Chinese Academy of Agricultural Sciences, Key Laboratory of Animal Parasitology of Ministry of Agriculture, Minhang, Shanghai, PR China; 2 Jiangsu Co-innovation Center for Prevention and Control of Important Animal Infectious Diseases and Zoonoses, Yangzhou, PR China; National Taiwan University, TAIWAN

## Abstract

*Eimeria tenella* is an obligate intracellular parasite that actively invades cecal epithelial cells of chickens. The basis of cell invasion is not completely understood, but some key molecules of host cell invasion have been discovered. This paper investigated the characteristics of calcium-dependent protein kinase 4 (*Et*CDPK4), a critical molecule in *E*. *tenella* invasion of host cells. A full-length *EtCDPK4* cDNA was identified from *E*. *tenella* using rapid amplification of cDNA ends. *EtCDPK4* had an open reading frame of 1803 bp encoding a protein of 600 amino acids. Quantitative real-time PCR and western blotting were used to explore differences in *EtCDPK4* transcription and translation in four developmental stages of *E*. *tenella*. *EtCDPK4* was expressed at higher levels in sporozoites, but translation was higher in second-generation merozoites. *In vitro* invasion inhibition assays explored whether *Et*CDPK4 was involved in invasion of DF-1 cells by *E*. *tenella* sporozoites. Polyclonal antibodies against recombinant *Et*CDPK4 (r*Et*CDPK4) inhibited parasite invasion, decreasing it by approximately 52%. Indirect immunofluorescence assays explored *Et*CDPK4 distribution during parasite development after *E*. *tenella* sporozoite invasion of DF-1 cells *in vitro*. The results showed that *Et*CDPK4 might be important in sporozoite invasion and development. To analyze *Et*CDPK4 functional domains according to the structural characteristics of *Et*CDPK4 and study the kinase activity of r*Et*CDPK4, an *in vitro* phosphorylation system was established. We verified that r*Et*CDPK4 was a protein kinase that was completely dependent on Ca^2+^ for enzyme activity. Specific inhibitors of r*Et*CDPK4 activity were screened by kinase activity *in vitro*. Some specific inhibitors were applied to assays of DF-1 cell invasion by *E*. *tenella* sporozoites to confirm that the inhibitors functioned *in vitro*. W-7, H-7, H-89, and myristoylated peptide inhibited DF-1 invasion by *E*. *tenella* sporozoites. The experimental results showed that *Et*CDPK4 may be involved in *E*. *tenella* invasion of chicken cecal epithelial cells.

## Introduction

The protozoan phylum Apicomplexa comprises thousands of obligate intracellular parasites, many of which cause significant human and animal health problems. For example, *Toxoplasma gondii* infects approximately one-third of the global human population and causes severe disease in immunocompromised patients and pregnant women [1]. Other examples are *Plasmodium falciparum*, the causative agent of malaria, which causes more than 1 million deaths per year [2] and *Eimeria* species, the protozoan parasites that cause the severe intestinal disease coccidiosis [3, 4].

Avian coccidiosis is a major disease of poultry caused by parasitic *Eimeria* species including *Eimeria tenella*, *Eimeria necatrix*, *Eimeria acervulina*, *Eimeria maxima*, *Eimeria brunetti*, *Eimeria mitis* and *Eimeria praecox*. Coccidiosis causes severe economic losses in the poultry industry every year [5]. *Eimeria* have complex life cycles and need to invade the intestinal epithelium of chickens to develop and propagate. The invasion of host gut epithelial cells by *Eimeria* species is a complex, multistep process that begins with the apical attachment of the parasite to the host cell. This is followed by rapid internalization to form an intracellular, parasitophorous vacuole (PV) that encloses the newly invaded parasite, enabling its survival within the host [6]. To perpetuate the infection, *Eimeria* need to egress from infected cells and then reinvade uninfected cells. In response to these events, parasites have developed regulatory mechanisms for self-proliferation and invasion. During these processes, specialized secretory organelles known as micronemes, rhoptries and dense granules deliver cargo proteins in a coordinated fashion. Secreted proteins are thought to be central to invasion and the establishment of infection [7, 8]. However, secretion by these organelles is controlled by intracellular calcium as a second messenger, which is important in signal transduction cascades, including for protein secretion, gliding motility, invasion of and egress from host cells, proliferation and differentiation [9].

In Apicomplexan parasites, calcium-dependent protein kinases (CDPKs) are main receptors of Ca^2+^ signals [10, 11]. CDPKs have been identified throughout the plant kingdom and in some protozoans, but not in animals or fungi [12]. CDPKs have two key domains, a Ser/Thr kinase domain and an EF-hand-type calcium-binding domain. They also contain an N-terminal variable domain, an auto-inhibitory junction region and a C-terminus [13]. The N-terminal domain shows the highest sequence divergence among CDPKs and often contains myristoylation or palmitoylation sites that are believed to be associated with subcellular targeting [14]. The C-terminal domain is also variable and differs in lengths and amino acid compositions among CDPKs. The N- and C-terminal variable domains are suggested to determine the specific function of individual CDPKs [15].

Increasing evidence suggests that CDPKs control important physiological events in Apicomplexan parasite life cycles. For example, conditional suppression of *T*. *gondii* CDPK1 (*Tg*CDPK1) weakens microneme protein secretion, parasite gliding motility, host cell invasion and egress ability [16, 17]. *Pb*CDPK4 from *Plasmodium berghei*, an ortholog of *Tg*CDPK1, regulates cell cycle progression in the male gametocyte [18]. Genetic disruption of *Tg*CDPK3 demonstrates that it has a regulatory function in parasite physiology in addition to the ionophore-induced egress process [19, 20, 21]. Apicomplexa parasites contain multiple *CDPK* genes; *Plasmodium* species possess seven [22], Billker *et al*. found that *T*. *gondii* contains 12 [23]. To our knowledge, only three *E*. *tenella* CDPK members (*Et*CDPK1, *Et*CDPK2 and *Et*CDPK3) have been studied [24, 25], and the physiological functions of most *Et*CDPKs remain unclear. Studies suggest that CDPKs regulate biological functions in Apicomplexa parasites. However, CDPKs regulatory mechanisms and targets remain unclear in Apicomplexa parasites. Nonetheless, this family of CDPKs has received attention as potential drug targets in Apicomplexan parasites.

Because the CDPKs have essential functions in Apicomplexan parasites and are absent in mammalian and avian hosts, CDPKs are promising targets for research on drugs against *Eimeria* species and related Apicomplexans parasites [26]. Some selective inhibitors against their kinase activity have been generated [27, 28].

We studied new members of the *E*. *tenella* CDPK family. We carried out a comprehensive analysis including the cloning, sequencing, protein expression and characterization of a novel *EtCDPK4* gene and protein. We provided novel insights into *E*. *tenella* invasion and development from a detailed study of the expression of *Et*CDPK4. This study aimed to provide information for further research and discovery of other members of the CDPK family of *E*. *tenella*.

## Materials and Methods

### Ethics Statement

This research with chickens was approved by the Shanghai Administration Committee of Laboratory Animals (GB14925-2010) and performed in accordance with the Chinese Academy of Agricultural Sciences Institutional Animal Care and Use Committee guidelines.

### *E*. *tenella* Propagation and Purification

The Shanghai strain of *E*. *tenella* was isolated from a sample collected from a chicken farm in Shanghai, China in 1985 and was maintained in our laboratory (Resource Number: CAAS21111601, Shanghai Veterinary Research Institute innovation team of protozoosis preservation, Chinese Academy of Agricultural Sciences). 60 healthy AA chickens were fed with coccidian-free water and feed. *E*. *tenella* was propagated as previously described [29] by passage through 2-week-old coccidian-free chickens. Unsporulated oocysts were obtained from the cecal contents of chickens at 8 days post-infection (p.i.). Some unsporulated oocysts was purified and stored in liquid nitrogen. The rest were incubated in 2.5% potassium dichromate to induce sporulation. After sporulation, oocysts were collected and purified. Sporozoites were prepared from cleaned, sporulated oocysts by excystation *in vitro*. Second-generation merozoites were collected from ceca at 120 h p.i. from chickens inoculated with 8.0 × 10^4^ sporulated oocysts per bird. Isolation was carried out as previously described [30]. Isolated sporozoites and second-generation merozoites were stored in liquid nitrogen.

The chicken embryo fibroblast cell line DF-1, derived from East Lansing Line (ELL-0) chicken embryos, was used for infection, inhibition assays and immunofluorescence experiments [31].

### Molecular cloning of the *EtCDPK4* full-length cDNA by RACE

Total RNA was extracted from *E*. *tenella* sporozoites using TRIzol reagent (TaKaRa, Tokyo, Japan) according to the manufacturer’s protocol. RNA quality was analyzed by 1% agarose gel electrophoresis and visualization with Ethidium Bromide staining. Total RNA concentration was quantified by UV spectrophotometry (Eppendorf, Hamburg, Germany). Rapid amplification of cDNA ends (RACE) was carried out with GeneRacer kits (Invitrogen, Carlsbad, CA, USA) to obtain the full-length 5' -and 3' -termini. RACE primers were designed based on gene sequences of the *CDPK* family (ETH_00010685, http://www.genedb.org/Homepage/Etenella). Approximately 10 μg of total RNA was used to synthesize 5' -and 3' -RACE-Ready cDNA. Sequences were amplified by Touchdown PCR with either 5'- RACE or 3'- RACE gene-specific primers (Table 1) and GeneRacer 5'- or 3'- primers. Nested PCRs were performed with nested gene-specific primers and 5'- or 3'- RACENEST primers (Table 1). Amplification products were subjected to electrophoresis using 1% agarose gels. Single bands were extracted, purified, cloned into the pGEM-T Easy vector (Promega, Madison, WI, USA), and propagated in *Escherichia coli* TOP10 (TIANGEN, Beijing, China) competent cells. Clones were sequenced by Shanghai Sunny Biotechnology Co., Ltd. Sequences of 5'-RACE and 3'-RACE were compared with the original *EtCDPK4* open reading frame (ORF) sequence using DNAstar software (Promega) to determine overlap. Full-length cDNA sequences were submitted to GenBank (Accession No. KU925778).

**Table 1:** Primer sequences used in this study.

Primer ID	Primer Sequences
5'-RACE Primer	5'-TCGAATATCTTCCGCAGGGCCGACTCA-3'
5'-RACENEST Primer	5'-CGACTCATCCACTGGGGGAAGCTGCAA-3'
3'-RACE Primer	5'-CGAAGCTGTCCTGGGCGGCGAAGACTA-3'
3'-RACENEST Primer	5'-GGCGATGGGCAGATTGACTGGGACGAA-3'
*Et*CDPK4UP	5'-CGGGATCCGAGCAGGTGATGGGTGGGCGGGAGGT-3'
*Et*CDPK4LOW	5'-GCGTCGACAATTCGTCCCAGTCAATCTGCCCAT-3'
*Et*CDPK4-RT(UP)	5'-GACCATCAGATTCACAAG-3'
*Et*CDPK4-RT(LOW)	5'-CTCAAAGACATCCACATC-3'
18Sr RNA sense	5'-TGTAGTGGAGTCTTGGTGATTC-3'
18Sr RNA antisense	5'-CCTGCTGCCTTCCTTAGATG-3'

### Bioinformatics analysis of *EtCDPK4*

The sequence of full-length *EtCDPK4* cDNA was analyzed. Signal peptide sequences, transmembrane and hydrophobic regions, genetically mobile domains and conservative structure predictions were identified using SignalP (http://www.cbs.dtu.dk/), TMPRED (http://www.ch.embnet.org/), Hydrophobic (http://web.expasy.org/), SMART (http://smart.embl-heidelberg.de/) and CDD (http://www.ncbi.nlm.nih.gov/Structure/) computational tools. Secondary structure, antigen index, flexible region and surface probability of *Et*CDPK4 were analyzed using DNAstar.

### Expression and Purification of Recombinant CDPK4 Proteins

Total RNA was extracted from sporozoites and the first-strand cDNA templates were generated by M-MLV reverse transcriptase (Invitrogen, Carlsbad, CA, USA) using oligo (dT) as primer. A 1660-bp (from 303 bp to 1962 bp) fragment of *EtCDPK4* encoding 550 amino acids with a calculated molecular weight of 62.6 kDa was amplified using primers *Et*CDPK4UP and *Et*CDPK4LOW according to the ORF sequence. Sequence-specific primers were designed to contain sites for *Bam* HI in the forward primer (*Et*CDPK4UP) and *Sal* I in the reverse primer (*Et*CDPK4LOW) (Table 1). PCR amplification was performed as follows program: 94°C for 5 min, 38 cycles 94°C for 90 s, 56°C for 45 s and 72°C 90 s, followed by 10 min at 72°C. After sequencing, fragments were digested with *Bam* HI and *Sal* I, purified from agarose gels by TIANgel Midi Purification Kits (TIANGEN) and ligated into the corresponding sites of the expression vector pCold I and sequenced. The pCold I-*EtCDPK4* plasmid was transformed into *E*. *coli* (BL21) to produce a recombinant protein (r*Et*CDPK4) with a 6×-His tag at the N- terminus. r*Et*CDPK4 protein was induced by 1.0 mM IPTG (Sigma, St. Louis, MO, USA) at 16°C. Induced bacterial cells were incubated for 24 h and harvested by centrifugation. Cell pellets were lysed by sonication and the insoluble portion of the pellet was suspended in 10 mM imidazole binding buffer and purified by His Bind Resin (Merck, Darmstadt, Germany). Yield of the affinity-purified protein was estimated using a Biophotometer (Eppendorf, Hamburg, Germany). Purified r*Et*CDPK4 protein was visualized by 12% SDS-PAGE. Then purified protein was stored in aliquots at– 20°C.

### Production of Anti-r*Et*CDPK4 Serum and Identification of r*Et*CDPK4

Two 2-month-old rabbits were immunized with 0.2 mg r*Et*CDPK4 emulsified in Freund Complete Adjuvant (Sigma) by intraperitoneal injection. Rabbits were boosted three times with Freund’s incomplete adjuvant at 2-week intervals. Eight days after final immunization, polyclonal antibody serum was separated from two rabbits blood.

r*Et*CDPK4 was resolved by 12% SDS-PAGE and transferred to PVDF membranes (Millipore, Bellerica, MA, USA). Western blots were performed according to standard procedures by using rabbit anti-merozoite protein sera (1:500) previously obtained in our lab or anti-*His*-tag monoclonal antibody (1:2000). Native rabbit IgG (1:1000) was the negative control. IRDye 800CW goat anti-rabbit IgG (LI-COR, Lincoln, NE, USA) and IRDye 680RD donkey anti-mouse IgG (1:25,000) (LI-COR, Lincoln, NE, USA) were used as the secondary antibody. Indirect Enzyme Linked Immunosorbent Assay (ELISA) was used to determine rabbit anti-r*Et*CDPK4 serum titers.

### *Et*CDPK4 Transcription and Translation Analysis in *E*. *tenella* Life Stages

Total RNAs isolated from four life stages of *E*. *tenella* (unsporulated oocysts, sporulated oocysts, sporozoites and second-generation merozoites) were treated with DNase I (Invitrogen) according to the protocol. Quality and quantity of total RNAs were assessed as described above. The first cDNAs were generated by SuperScript II reverse transcriptase (Invitrogen) using random primers. Quantitative real-time PCR (qRT-PCR) was performed on an Eppendorf Mastercycler ep Realplex (Eppendorf, Hamburg, Germany) using the SYBR1 green I dye method. Negative (no template) controls were included. A fragment encoding the *E*. *tenella* 18S ribosomal RNA was used as a control. Reactions were carried out in triplicate and experiments were performed six times. Primers for real-time PCR are in Table 1. Primers for *EtCDPK4* (*Et*CDPK4-RT[UP] and *Et*CDPK4-RT[LOW]) and 18S rRNA were designed by the Beacon Designer program (Corbett Robotics, USA). Relative mRNA expression level was determined as the ratio of *EtCDPK4* to 18S rRNA.

To investigate expression of *Et*CDPK4 in developmental stages, lysates from *E*. *tenella* unsporulated oocysts, sporulated oocysts, sporozoites and second-merozoites were prepared using cell lysis buffer for western Blot and IP (Beyotime, Haimen, China). Protein concentrations were determined using BCA Protein Assay kits (Beyotime) and separated on 12% SDS-PAGE. Western blots were performed according to standard procedures [32]. Anti-r*Et*CDPK4 antibodies were used at 1:100 and mouse monoclonal anti-α-tubulin antibodies (Sigma) at 1:1000 were controls. Secondary antibodies were used as above. IRDyes were detected using an ODYSSEY Infrared Imaging System (LI-COR).

### Assays for r*Et*CDPK4 protein kinase activity

To calculate r*Et*CDPK4 activity units in catalytic reactions, we defined one unit r*Et*CDPK4 activity as a nanomole of phosphate group transferred to a substrate per minute per milliliter. *In vitro* phosphorylation reactions were performed using Non-radioactive PepTag assays (Promega). Assays (25 μL) were carried out in 20 mM HEPES-KOH, pH 7.4, 1.3 mM CaCl_2_, 1 mM DTT, 10 mM MgCl_2_, 1 mM ATP, 5 μg sonicated phosphatidylserine, 1 mM phenylmethylsulfonyl fluoride, 5 ng leupeptin, 5 ng aprotinin, 2 mM mercaptoethanol, 0.05% Triton X-100, 47.5 μM PepTag^**®**^C1-Peptide, 2 μL peptide protective solution, with an aliquot of purified r*Et*CDPK4 (10 μg, initial concentration about 1.0 mg/mL). Phosphorylation reactions were performed for 30 min at 30°C and stopped by heating at 95°C for 15 min. Phosphorylated C1 peptides were separated from non-phosphorylated peptides by electrophoresis on 0.8% agarose gels at 140 V for 30 min. To estimate the amount of phosphorylated peptide, bands were excised from gels under UV light, melted at 95°C and mixed (325 μL) with gel solubilization solution (75 μL, Promega) and glacial acetic acid (100 μL). Optical absorbance values of solutions were measured with a NanoDrop2000/2000C spectrophotometer at 570 nm. r*Et*CDPK4 enzyme activity was measured using Beer’s Law as described above.

### Effect of Ca^2+^ Concentrations on r*Et*CDPK4 Activity

Based on r*Et*CDPK4 kinase activity assays, the initial reaction system was improved. The kinase activity assay had two portions: qualitative and quantitative detection. Assays (30 μL) were carried out in 20 mM HEPES-KOH, pH 7.4, 1 mM DTT, 10 mM MgCl_2_, 1 mM ATP, 5 μg sonicated phosphatidylserine, 1 mM phenylmethylsulfonyl fluoride, 5 ng leupeptin, 5 ng aprotinin, 2 mM mercaptoethanol, 0.05% Triton X-100 (v/v), 47.5 μM PepTagC1-Peptide, 2 μL peptide protective solution, and an aliquot of purified r*Et*CDPK4 (10 μg, initial concentration about 1 mg/mL), and five concentrations of CaCl_2_: 0 μM, 10 μM, 50 μM, 100 μM, or 1000 μM. Negative controls and r*Et*CDPK4 sample controls were used. Phosphorylation reactions were for times and temperatures as above. Phosphorylated-C1-peptides were separated and amounts of phosphorylated peptide estimated as above. r*Et*CDPK4 kinase activity was measured by spectrophotometer and experiments were performed twice.

### Screening and Analysis of r*Et*CDPK4 Specific Inhibitors

By bioinformatics analysis of *Et*CDPK4 functional domains, we chose seven inhibitors: W-7 (Sigma), H-7 (Sigma), H-89 (Beyotime), staurosporine (Beyotime), D-sphingosine (Sigma), Ro-31-8220 (Sigma) and myristoylated peptide (Promega). These inhibitors belonged to three categories: ATP competitive inhibitor, hypothetical-substrate inhibitor and Ca^2+^-binding-domain inhibitor. Staurosporine, H-7, H-89, W-7 and Ro-31-8220 belong to the category of ATP competitive inhibitor; Myristoylated peptide belongs to the hypothetical-substrate inhibitor; D-sphingosine belongs to the Ca^2+^-binding-domain inhibitor. Inhibitory concentrations were as recommended by the supplier instructions. The inhibitors’ final concentrations were adjusted to 100 μM in the reaction system. Assays (30 μL) were carried out in 20 mM HEPES-KOH, pH 7.4, 1.3 mM CaCl_2_, 1 mM DTT, 10 mM MgCl_2_, 1 mM ATP, 5 μg sonicated phosphatidylserine, 1 mM phenylmethylsulfonyl fluoride, 5 ng leupeptin, 5 ng aprotinin, 2 mM mercaptoethanol, 0.05% Triton X-100, 47.5 μM PepTag C1-Peptide, 2 μL peptide protective solution, and purified r*Et*CDPK4 as above and specific inhibitor. Negative controls and r*Et*CDPK4 samples without inhibitors were included. Phosphorylation reactions, separation of phosphorylated-C1-peptides and estimation of amounts were as above. Experiments were performed three times. Data differences among groups were analyzed by one-way analysis of variance (ANOVA) Duncan test.

### Inhibition of DF-1 Invasion by Anti-r*Et*CDPK4 Polyclonal Antibody or r*Et*CDPK4 Specific Inhibitors

The chicken embryo fibroblast cell line DF-1 was used for inhibition assays [33]. Antibodies were purified with Protein A+G Agarose (Beyotime). *E*. *tenella* sporozoites were labeled using carboxyfluorescein diacetate, succinimidyl ester (CFDA SE, Beyotime) according to the manufacturer’s instructions. Labeled sporozoites (1.0 **×** 10^8^) were resuspended in 1 mL CM (Gibco, Grand Island, NY, USA), purified anti-r*Et*CDPK4 polyclonal antibody, IgG from native rabbit serum (negative control), or an equivalent volume of PBS (normal control) was added to labeled sporozoites to final concentrations of 50, 100, 200, 300 or 400 μg/mL respectively. Sporozoites were incubated at 37°C for 2 h, washed twice in sterile phosphate buffered saline, then infected 2.0 **×** 10^5^ DF-1 cells in 24-well plates (Corning, NY, USA). After cultured 16 h at 41°C, cells were collected and analyzed by flow cytometry (Beckman Coulter, USA). Uninfected DF-1 cells were the control. Infected cells, uninfected cells, and free sporozoites were gated using RXP software (Beckman Coulter, USA) to count infected (labeled sporozoites) and uninfected (fluorescence-free) cells. All of invasion-inhibition assays were performed in triplicate. Percentages of cells infected in the presence or absence of anti-r*Et*CDPK4 polyclonal antibody were used to calculate inhibition rate, as previously described: inhibition = 100% × (1− [% (infected cells^Antibody treatment^) /% (infected cells^negative control^)]) [34].

*E*. *tenella* sporozoites were labeled as above. Labeled sporozoites (4.0 **×** 10^8^) were resuspended in 1 mL of DMEM and incubated with specific inhibitors of r*Et*CDPK4 (100 μM) or dimethyl sulfoxide (DMSO; negative control) (100 μM) for 2 h at 37°C. All assays were performed in triplicate. Uninfected DF-1 cells were controls. Infected cells, uninfected cells, and free sporozoites were gated as above to count infected (labeled sporozoites) and uninfected (fluorescence-free) cells. Percentages of infected cells in the presence or absence of r*Et*CDPK4 specific inhibitors were used to calculate inhibition rates as described.

### Indirect Immunofluorescence Assays of *Et*CDPK4 Expression During First Schizogony

DF-1 cells were infected with sporozoites at 41°C, 5% CO_2_ at one sporozoite per cell in DMEM (Gibco) supplemented with 5% FBS, 100 U/mL penicillin/streptomycin, 2 mM L-glutamine. Purified freshly excysted sporozoites were incubated in PBS or complete medium for 2 h at 41°C and washed before transferring to a glass slide and air-dried as previously described [35, 36]. Sporozoites incubated in CM for 2 h at 41°C were used to infect cells. After infection 12, 24, 36, 48, 60 and 72 h, cells were collected and washed before transferred to glass slides and air-dried. Slides were fixed in 2% paraformaldehyde in PBS for 30 min and permeabilized using 1% Triton X-100 in PBS for 30 min. Slides were blocked with PBS containing 2% (w/v) bovine serum albumin at room temperature for 2.5 h. A 1:500 dilution of anti-r*Et*CDPK4 polyclonal antibody was added and incubated for 2 h at 37°C. Then a 1:500 dilution of a goat anti-rabbit IgG fluorescein isothiocyanate (FITC)-conjugated antibody (Sigma-Aldrich, St. Louis, State of Missouri, USA) was added and incubated for 2.5 h at 37°C. Nuclei were stained with 15 μg/mL 4, 6-diamidino-2-phenylindole (DAPI) (Beyotime) at room temperature for 10 min. After each step, slides were washed six times with PBS containing 0.5% (v/v) Tween-20. Finally, slides were mounted using 100 μL Fluoromount Aqueous Mounting Medium (Sigma-Aldrich). Before observation under a fluorescence microscope (Olympus, Tokyo, Japan), 50 μL 1, 4—diazabicyclo [2. 2. 2] octane (DABCO; Sigma) was added.

### Statistical Analysis

Statistical analysis was performed using Microsoft Office Excel for Windows version 2013 (Redmond, Washington, USA) and GraphPad Prism 5.0 (GraphPad, La Jolla, CA, USA). All data, including *Et*CDPK4 Transcription and Translation Analysis in *E*. *tenella* Life Stages, Inhibition of DF-1 Invasion by Anti-r*Et*CDPK4 Polyclonal Antibody or r*Et*CDPK4 Specific Inhibitors, Effect of Ca^**2+**^ Concentrations on r*Et*CDPK4 Activity, Screening and Analysis of r*Et*CDPK4 Specific Inhibitors, were analyzed. Differences among groups were tested by one-way analysis of variance (ANOVA) Duncan test. The data are presented as mean ± standard deviation (SD). P < 0.05 was considered significant and P < 0.01 highly significant.

## Results

### Characteristics of *EtCDPK4* Sequence by Bioinformatics

**Fig 1:**
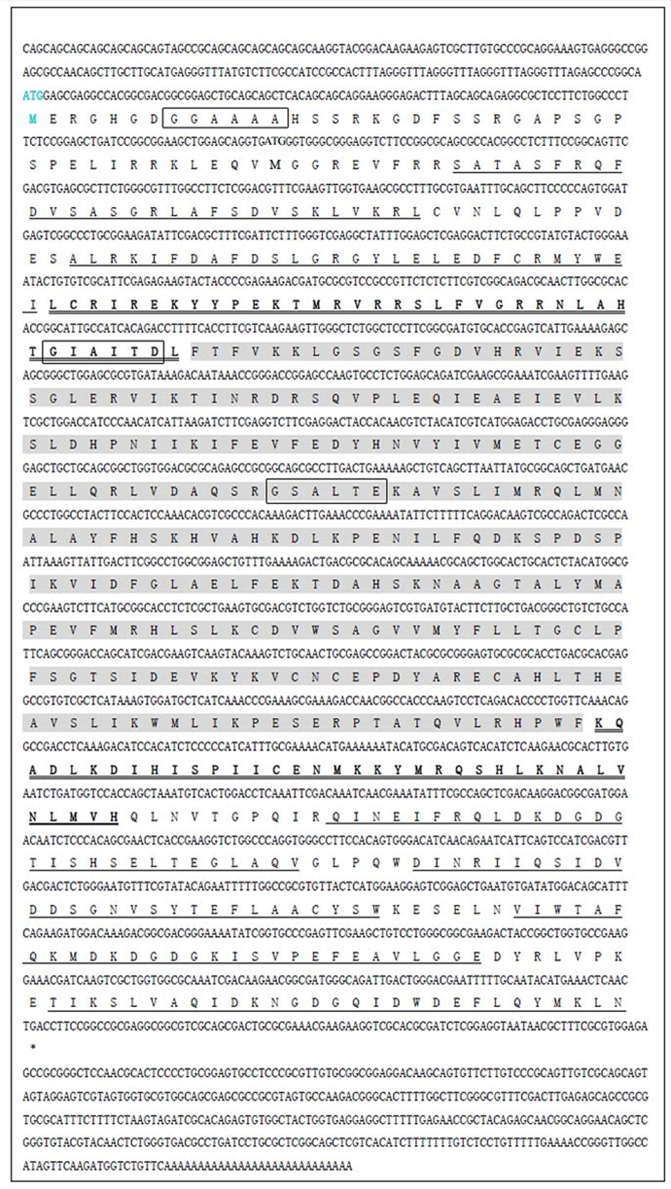
Complete sequence of *EtCDPK4* cDNA and deduced amino acids. Single underlined, six calcium-binding EF-hand motifs; Double underlined, junction domain; Shaded, serine/threonine kinase domain; Rectangular box, N-myristoylation site.

The full-length *EtCDPK4* cDNA was 2499 bp with a single ORF of 1803 bp (positions 186 bp– 1988 bp) encoding a polypeptide of 600 amino acid residues with a calculated molecular mass of approximately 68.3 kDa. Fig 1 shows the complete nucleotide sequence of *EtCDPK4* and the deduced amino acids. The cDNA contained a 5'- untranslated region (UTR) of 185 bp and a 3'- UTR of 511 bp. The 3'- UTR contained a characteristic poly A tail (AAAAAA) but without a classic polyadenylation signal (AATAAA).

**Fig 2:**
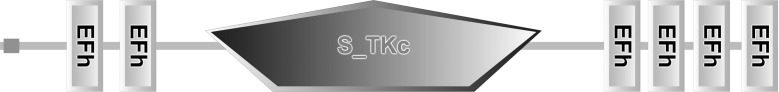
Genetically mobile domain and conservative structure analysis of the *Et*CDPK4. S_TKc, Serine/Threonine protein kinases, catalytic domain; EFh, calcium binding motif.

TMPRED analysis of the transmembrane regions of the *Et*CDPK4 amino acid sequence found a transmembrane region in the Val302—Ile^324^ position. SignalP analysis revealed that the protein most likely does not contain a signal peptide. Structural module and conservative structure predictions indicated that the protein contained distinct domains characteristic of a calcium-dependent protein kinase, including an amino-terminal kinase domain with a typical serine/threonine-kinase active site, a carboxy-terminal calmodulin-like domain with four EF-hand motifs and an amino-terminal calmodulin-like domain with two EF-hand motifs for calcium-binding (Fig 2). Also predicted were three N-myristoylation sites and two N-glycosylation sites. The gene was designated *EtCDPK4* (GenBank Accession No. KU925778). DNAstar analysis of the secondary structure, antigen index, flexible regions and surface probability of *Et*CDPK4 showed a polypeptide rich in α-helix, β-sheet, β-turn, random coil and flexible regions that might contribute to polypeptide chain folding to tertiary structures. The polypeptide antigen-index score was relatively high at 1.7. Therefore, the *Et*CDPK4 polypeptide had antigenic sites and was predicted to be immunogenic. Surface probability analysis of the *Et*CDPK4 polypeptide had high scores of about 6.0, suggesting that the function of the *Et*CDPK4 was involved in the *E*. *tenella* membrane surface.

### Expression and Purification of Recombinant CDPK4

**Fig 3:**
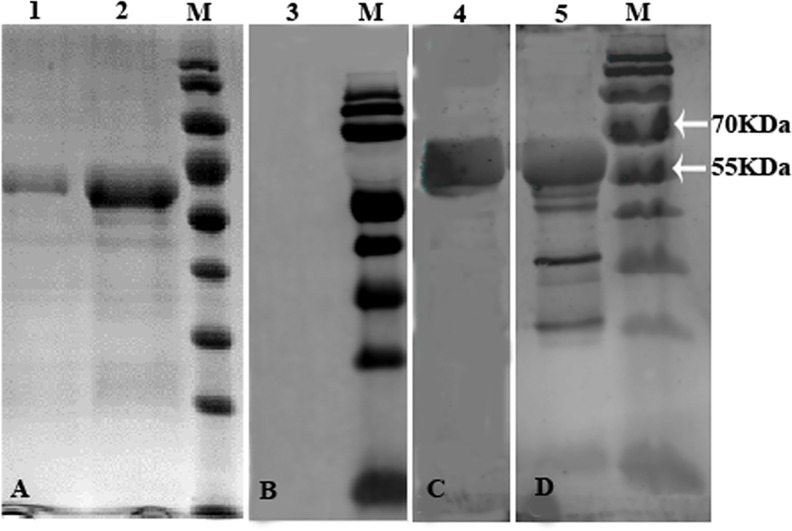
Rabbit anti-serum against *E*. *tenella* second merozoites or anti-His monoclonal as the primary antibody. Reactive bands were visualized using IRDye 800CW goat anti-rabbit IgG and IRDye 680RD donkey anti-mouse IgG. M, protein standard molecular weight (15.0 kDa to 170.0 kDa). A, Lane 1, imidazole elution buffer 150 mM; elution of r*Et*CDPK4 proenzyme. Lane 2, imidazole elution buffer 250 mM; elution of r*Et*CDPK4 proenzyme; B, Lane 3, native rabbit serum IgG. C, Lane 4, anti-His tag monoclonal antibody. D, Lane 5, rabbit anti-merozoites whole serum.

The 1660-bp fragment (location: 118 bp—1777 bp) of the *EtCDPK4* ORF was amplified by RT-PCR according to the structural and functional domains, because it is difficult to amplify the full-length of ORF of *EtCDPK4*. The protein encoded by this fragment contained all distinct domains, including serine/threonine-kinase active site, EF-hand motifs for calcium-binding. Then the fragment was ligated with expression vector pColdⅠto create an expression plasmid pCold-*EtCDPK4*. After sequencing, pCold-*EtCDPK4* was transformed into *E*. *coli* BL21 to induce expression of recombinant protein. Electrophoresis results showed that r*Et*CDPK4 was a soluble protein. Lysates of *E*. *coli* included a band on SDS-PAGE gels with a molecular weight (MW) close to the theoretical 62.6 kDa MW of *Et*CDPK4. r*Et*CDPK4 was purified using column affinity chromatography and identified by SDS-PAGE (Fig 3A).

### Production of Anti-r*Et*CDPK4 and Identification of r*Et*CDPK4

Serum was generated using rabbits. Western blots showed that r*Et*CDPK4 was recognized by rabbit anti-merozoites serum or anti-His tag monoclonal antibody (Fig 3B, 3C and 3D). The rabbit anti-r*Et*CDPK4 serum titer was 1:12, 800 by indirect ELISA.

### *EtCDPK4* Transcripts and Protein in *E*. *tenella* Developmental Stages

**Fig 4:**
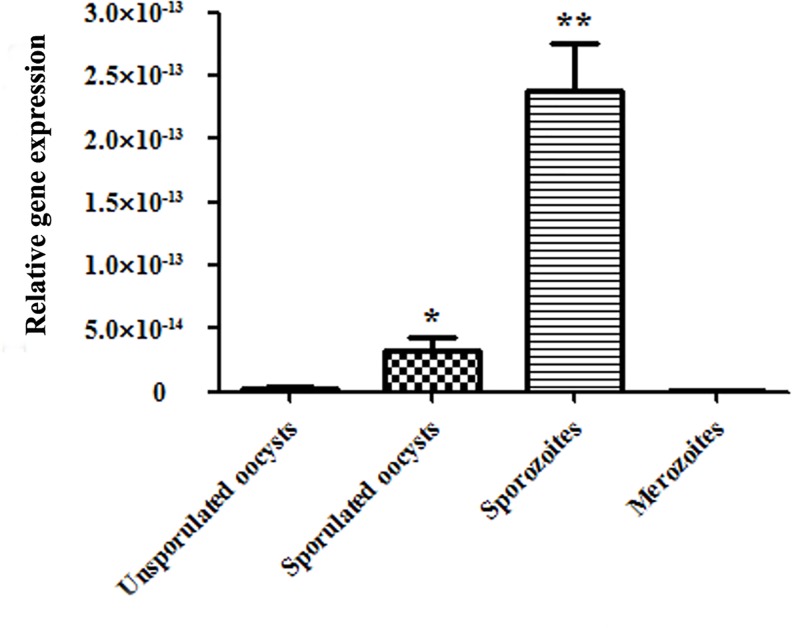
*EtCDPK4* transcription at different *E*. *tenella* life stages. Bars not sharing the same symbol were significantly different (P < 0.05) and the error bars indicate standard deviations.

To determine the mRNA level of *EtCDPK4* in unsporulated oocysts, sporulated oocysts, sporozoites, and second-generation merozoites of *E*. *tenella*, total RNA was subjected to qPCR analysis. Among the four development stages, *EtCDPK4* mRNA was highest in sporozoites; transcripts were nearly undetectable in unsporulated oocysts and merozoites (Fig 4).

**Fig 5:**
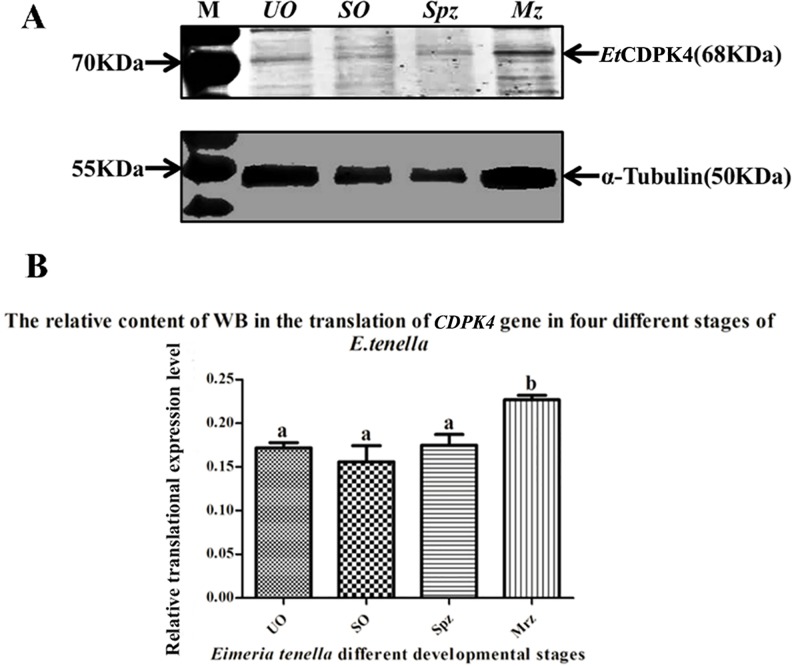
Analysis of western blots of *Et*CDPK4 at different of *E*. *tenella* stages probed with anti-r*Et*CDPK4. A, qualitative analysis of *Et*CDPK4 at different stages of *E*. *tenella* by western blot; α-tubulin, internal reference protein. B, relative quantitative analysis of *Et*CDPK4 expression difference at different stages of *E*. *tenella*. Lanes: UO, unsporulated oocysts; SO, sporulated oocysts; Spz, sporozoites; Mz or Mrz, second-generation merozoites. Bars with different letters were significantly different (P < 0.05) and the error bars indicate standard deviations. WB, western blot. M, protein weight standard (from top to bottom: 170 kDa, 130 kDa, 100 kDa, 70 kDa, 55 kDa, 40 kDa, 35 kDa, 25 kDa, 15 kDa).

The presence of *Et*CDPK4 protein in the four developmental stages was determined by immunoblotting using antibodies from rabbits immunized with r*Et*CDPK4. Anti-α-tubulin monoclonal antibodies were used as internal reference controls. Anti-r*Et*CDPK4 labeled the same 68.3-kDa band in second-generation merozoites, unsporulated oocysts and sporozoites with weak reactivity in sporulated oocysts (Fig 5A). Thus, the *Et*CDPK4 had a high expression level in stage of the second-generation merozoites, while the *Et*CDPK4 in other three developmental stages of the sporozoites, unsporulated oocysts and sporulated oocysts were approximately at the same level (Fig 5B).

### Protein Kinase Activity of r*Et*CDPK4

**Fig 6:**
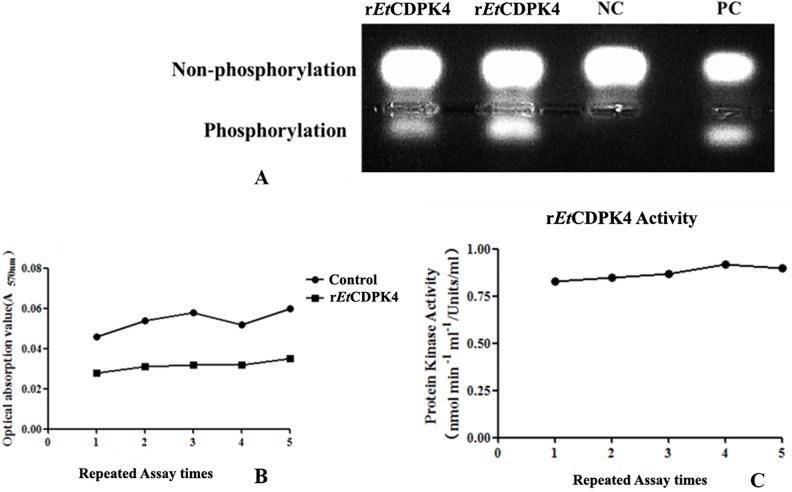
r*Et*CDPK4 protein kinase activity assays. A, Qualitative detection of r*Et*CDPK4 kinase activity. NC, negative control (no r*Et*CDPK4); PC, positive control (PKC). B, Quantitative detection of five times the optical absorption value (A_**570nm**_ value). C, Quantitative detection of r*Et*CDPK4 kinase activity. Phosphorylation, the gel band that the PepTag^**®**^C1-Peptide was phosphorylated by r*Et*CDPK4. Non-phosphorylation, the gel band that the remainder PepTag^**®**^C1-Peptide was not phosphorylated by r*Et*CDPK4.

r*Et*CDPK4 was purified by column affinity chromatography (Fig 3A) and protein kinase activity was measured based on nonradioactive detection of *in vitro* phosphorylation of a PKC-specific fluorescent synthetic peptide substrate. Phosphopeptides were detected under UV light and quantified spectrophotometrically (Fig 6A, 6B and 6C). r*Et*CDPK4 had protein kinase activity with a reaction rate of about 0.87 nmol•min^-1^ •mL^-1^ protein or 0.87 units/mL.

### Effect of Ca^2+^Concentration on r*Et*CDPK4 Activity

**Fig 7:**
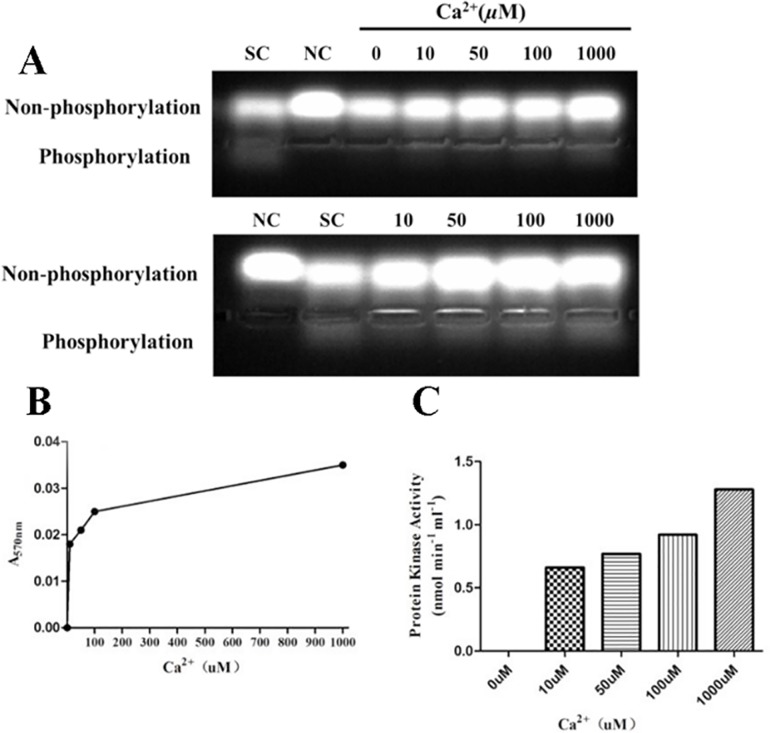
Kinase activity dependence on Ca^2+^ concentration by qualitative assays. SC, r*Et*CDPK4 sample control; NC, negative control. Five Ca^2+^ concentrations were: 0, 10, 50, 100, and 1000 μM. A, Qualitative detection assay; B, A_570nm_ value of phosphorylated short peptides at indicated calcium concentrations; C, r*Et*CDPK4 enzyme activity under indicated Ca^2+^ concentrations. Phosphorylation, the gel band that the PepTag^**®**^C1-Peptide was phosphorylated by r*Et*CDPK4. Non-phosphorylation, the gel band that the remainder PepTag^**®**^C1-Peptide was not phosphorylated by r*Et*CDPK4.

Enzyme activity was strongly dependent on Ca^2+^ concentrations with a calculated K_a_ of 10–1000 μM based on incubating r*Et*CDPK4 with various Ca^2+^ concentrations for *in vitro* phosphorylation assays (Fig 7A, 7B and 7C). With no Ca^2+^, r*Et*CDPK4 was inactivated and no substrate was phosphorylated. With increasing Ca^2+^ concentrations, the activity of r*Et*CDPK4 was enhanced in spite of the band relatively weak.

### Analysis of r*Et*CDPK4 Specific Inhibitors

**Fig 8:**
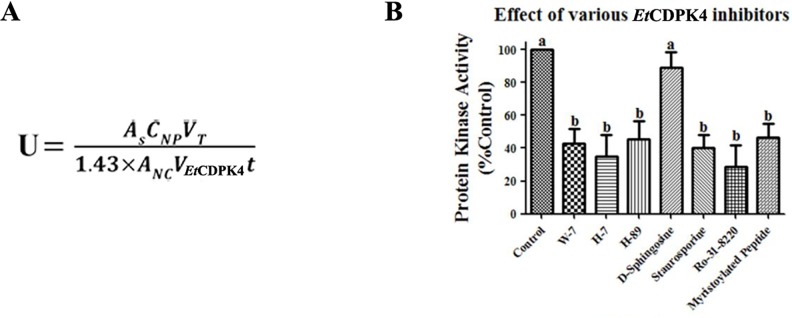
Quantitative analysis for seven inhibitors in r*Et*CDPK4 activity assays. A, formula for calculating r*Et*CDPK4 activity, r*Et*CDPK4 enzyme activity units were nmol×min^-1^×mL^-1^. A_S_, optical absorption for sample at 570 nm wavelength; C_NP_, standard concentration of PepTag^**®**^-C1; V_T_, total volume of samples in cuvette (500 μL); 1.43, correction coefficient for measuring absorbance (250/175); A_NC_, 570 nm optical absorption value of non-phosphorylated peptides in the negative control; V_*Et*CDPK4_, total volume of r*Et*CDPK4 in the reaction system (10 μL); t, time of phosphorylation reaction (30 min). B, Bars with different letters were significantly different (P < 0.05) and the error bars indicate standard deviations.

Seven inhibitors were applied to r*Et*CDPK4 activity assays with quantitative detection (Fig 8B). The activity of r*Et*CDPK4 in the reaction system with different inhibitors was detected by measuring the absorbance value of the cutting gel band at the 570 nm. The results showed that the absorbance values of phosphorylation reaction products treated by W-7, H-7, H-89, staurosporine, Ro-31-8220 and myristoylated peptide were lower than the sample control, but the absorbance value of phosphorylation reaction products treated by D-sphingosine was very close to the sample control. The calculation formula of kinase activity was showed in Fig 8A. Calculations for r*Et*CDPK4 activity showed that W-7, H-7, H-89, staurosporine, Ro-31-8220 and myristoylated peptide significantly decreased enzyme activity, while D-sphingosine had no effect.

### *Et*CDPK4 Localization During *In Vitro* Infection by Immunofluorescence

**Fig 9:**
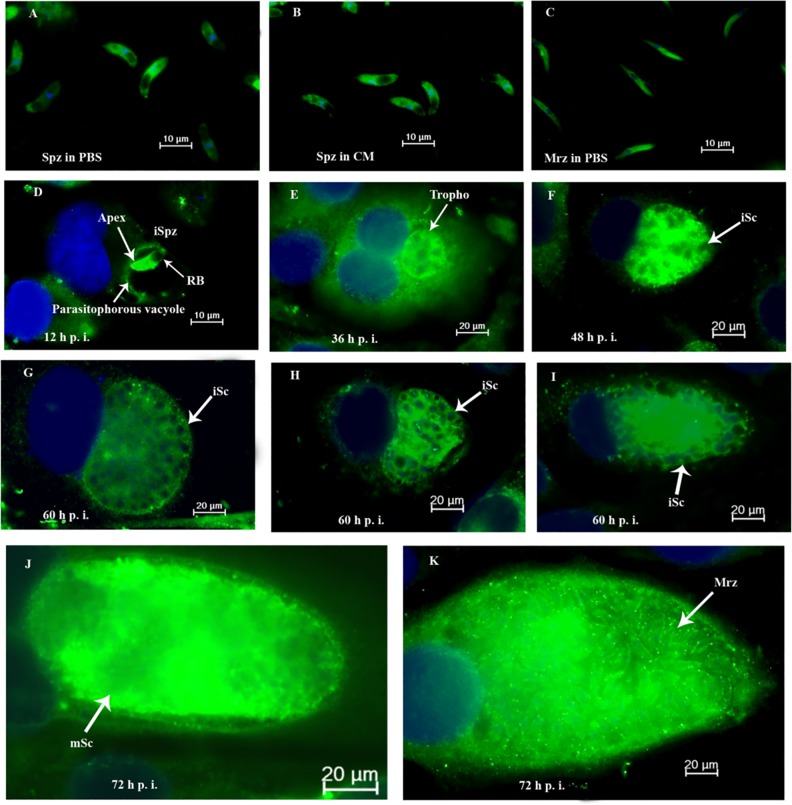
*Et*CDPK4 localization in DF-1 cell infection by immunofluorescence. Parasites were immune-stained with anti-r*Et*CDPK4 and visualized with FITC (green) and counterstained with DAPI (blue). A, Sporozoites (Spz) were incubated in PBS for 2 h or B, complete medium (CM) at 41°C. C, second-generation merozoites (Mrz) incubated in PBS for 2 h. Infected DF-1 cells were collected at indicated time points p.i. D, 12 h p.i. intracellular sporozoites (iSpz); E, 36 h p.i. intracellular trophozoite (Tropho); F, 48 h p.i. immature schizont (iSc); G, H, I, 60 h p.i. immature schizont (iSc); J, 72 h p.i. mature schizont (mSc); K, 72 h p.i., first-generation merozoites (Mrz) released from mature schizont.

Anti-r*Et*CDPK4 was used to localize *Et*CDPK4 in sporozoites and during first schizogony. *Et*CDPK4 exhibited a homogenous distribution pattern throughout the cytoplasm of sporozoites except for the refractive body and second-generation merozoites incubated in PBS for 2 h (Fig 9A and 9C). In contrast, when sporozoites were incubated in culture medium, *Et*CDPK4 expression did not change significantly (Fig 9B). After sporozoites invaded host cells, *Et*CDPK4 was mainly localized to the cytoplasm of parasites, except for the refractive body. Green fluorescence intensity was enhanced at this phase. Foci of intense *Et*CDPK4 staining were closely associated with the parasitophorous vacuole membrane (Fig 9D, 9H and 9J). Observations at 36 h p.i. showed that *Et*CDPK4 protein increased and was distributed in trophozoites (Fig 9E). Labeled *Et*CDPK4 was eventually uniformly dispersed in immature and mature schizonts and decreased in immature schizonts (Fig 9F, 9G, 9H, 9I and 9J). After formation of first-generation merozoites from mature schizonts in DF-1 cells, labeling increased (Fig 9K).

### Anti-r*Et*CDPK4 and r*Et*CDPK4 Specific Inhibitors Inhibited DF-1 Cell Invasion

**Fig 10:**
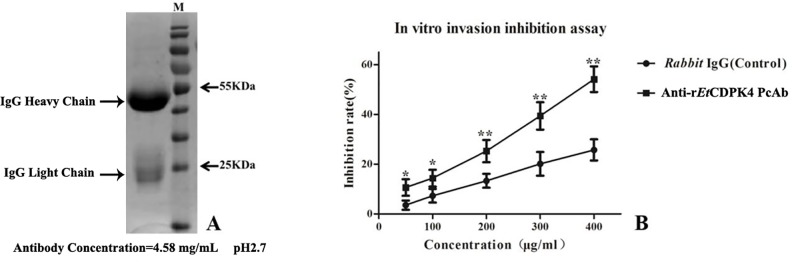
Inhibition of sporozoite invasion *in vitro*. A, Purification of rabbit anti-r*Et*CDPK4 serum by SDS-PAGE. B, Inhibition by anti-r*Et*CDPK4 serum and rabbit IgG. All assays were performed in triplicate. Anti-r*Et*CDPK4 PcAb, rabbit antiserum generated against r*Et*CDPK4 protein; rabbit IgG, IgG from native rabbit serum. The symbol “**” representability P < 0.01 and the “*” representability P < 0.05 for comparison of treatment with antibody against r*Et*CDPK4 and native rabbit serum at the same IgG concentration and the error bars indicate standard deviations. M, protein weight standard (from top to bottom: 170 kDa, 130 kDa, 100 kDa, 70 kDa, 55 kDa, 40 kDa, 35 kDa, 25 kDa, 15 kDa).

To evaluate *Et*CDPK4 effects on invasion of DF-1 cells by *E*. *tenella* sporozoites, invasion inhibition assays were performed. Protein function was blocked by pre-incubation of sporozoites with purified antibody against r*Et*CDPK4 (Fig 10A) before DF-1 cell infection. Ab*Et*CDPK4 inhibited invasion to 52% at an antibody concentration of 400 μg/mL, compared to infection with non-treated sporozoites. Inhibition was dose dependent. By comparison, native rabbit-sera IgG did not have a significant effect on invasion by *E*. *tenella* sporozoites (Fig 10B).

**Fig 11:**
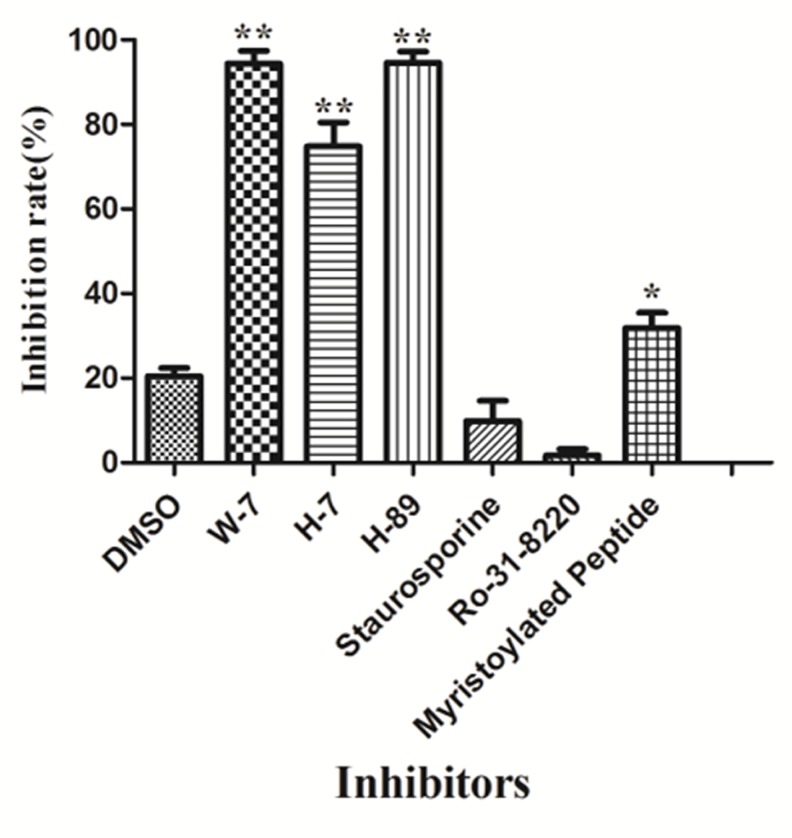
Inhibition of DF-1 invasion by *E*. *tenella* sporozoites using specific inhibitors of r*Et*CDPK4. The symbol “**” representability P < 0.01, very significant difference; “*” representability P < 0.05, significant difference. And the error bars indicate standard deviations.

Flow cytometry used to determine the effect of specific inhibitors on labeled sporozoites is in Fig 11. W-7, H-7, H-89 and myristoylated peptide significantly decreased invasion, while staurosporine and Ro-31-8220 had no effect.

## Discussion

In this work, the calcium-dependent protein kinase 4 of *E*. *tenella* was cloned and identified. The full-length cDNA of *EtCDPK4* was 2499 bp, with a 1803 bp ORF encoding a protein of 600 amino acids. The 5'-UTR was 185 bp. The 3'-UTR of 511 nucleotides ended with a poly (A) tail. Sequence analysis indicated that the protein contained domains characteristic of CDPKs: an N-terminal protein kinase domain, a C-terminal calmodulin-like domain with four calcium-binding EF-hand motifs, an N-terminal calmodulin-like domain of two calcium-binding EF-hand motifs, a junction domain and a very short N-terminal variable region. Searching the *E*. *tenella* genome database, the deduced amino acid sequence had 100% identity with a gene (ETH_00010685) encoding a putative CDPK. These results suggested that *Et*CDPK4 was a member of the *E*. *tenella* CDPK family.

CDPKs, encoded by multigene families, are widespread in plants and some Apicomplexan parasites. However, only four genes encoding for putative CDPKs of *E*. *tenella* are in the *E*. *tenella* genome database [24], including *EtCDPK4*. According to the number of calcium-binding EF-hand motifs, Apicomplexan parasite CDPKs are classified into four major categories [23]. The first category contains proteins with canonical CDPK structures containing four C-terminal EF-hand motifs. The second category contains proteins with three C-terminal EF hands. The other two groups of CDPKs have one or more N-terminal EF hands followed by a Ser/Thr kinase domain and three or four C-terminal EF-hand motifs. Motif scan results classified *Et*CDPK4 in the other group of canonical Apicomplexan parasite CDPKs. The EF-hand motif structure of *Cryptosporidium parvum* CDPK6 was also similar to *Et*CDPK4 [37]. The arrangement of the N-terminal EF domains is unusual and their role in the regulation of kinase activity has not been examined. Bioinformatic analysis on *Et*CDPK4 showed that it has three N-myristoylation sites. Several canonical CDPKs in parasites contain consensus motifs for N-myristoylation or palmitoylation, a feature also seen in plant CDPKs, many of which show membrane localization [38, 39]. This result suggests that association with membranes may be important in localization, as has been shown for *Pf*CDPK1, which is modified by both palmitate and myristate [40]. The role of such modifications in regulation of activity has not been explored in parasites.

The mRNA and protein levels of *Et*CDPK4 were examined in four developmental stages of *E*. *tenella*. Results from qPCR showed that mRNA for *EtCDPK4* was highest in the sporozoites and the lowest in the unsporulated oocysts and second-generation merozoites. These results showed that the *EtCDPK4* gene was transcribed predominantly at a distinct phase of the *E*. *tenella* life cycle. However, western blots showed that protein levels were highest for *Et*CDPK4 in second-generation merozoites and weakest in sporulated oocysts. This difference may be the result of underlying molecular mechanisms and signaling pathways in the four stages of *E*. *tenella* and should be investigated in more detail in future studies [41].

Previous studies of Apicomplexans parasites found that each *CDPK* gene is expressed predominantly at a distinct phase of the parasite life cycle. For example, another *CDPK* isoform of *E*. *tenella*, *Et*CDPK1, is expressed in sporulated oocysts, sporozoites and merozoites, but not in unsporulated oocysts, as determined by western blots [25]. *Et*CDPK3 has the highest expression in sporozoites by qPCR or western blots compared with other stages [24]. In *P*. *falciparum*, *Pf*CDPK1 is mainly expressed in the asexual blood stages of the parasite, particularly in late-stage schizonts [40]. In contrast, *PfCDPK3* is expressed specifically in the sexual erythrocytic stage [42]. *Pf*CDPK4 is detected only in *P*. *falciparum* gametocytes [32]. In the Apicomplexan parasite *T*. *gondii*, *Tg*CDPK1 and *Tg*CDPK3 are produced in the tachyzoite stage, but *Tg*CDPK2 protein is not detectable in this stage [43, 44].

In our study, mRNA for *EtCDPK4* was highest in sporozoites, but *Et*CDPK4 protein was higher in second-generation merozoites than in the other three stages. A high number of transcripts does not necessarily indicate corresponding amounts of translated protein, which is related to gene functions in the various the parasite stages [37]. Nonetheless, we propose a biological significance for CDPK4 in the *E*. *tenella* life cycle. Sporulated oocysts in the environment require material for metabolism from the storage of oocysts [45]. Without an energy supply, sporulated oocysts maintain a low metabolic rate until they can infect fresh host cells [46]. However, during this period, sporozoites are present in sporulated oocysts, and the energy required by sporozoites is provided by oocysts, so metabolism is moderate [47, 48]. To adapt to this mechanism, physiological activity of sporozoites is regulated at the gene level [49]. Protein translation consumes more energy in sporozoites, therefore mRNA for *EtCDPK4* was significantly higher in the sporozoites stage. When the merozoite stage invades the host cell and obtains nutrients, merozoites are in schizogony. In this period, the merozoites have higher metabolism and translation is activated [50], so merozoites can invade cells for schizogony and gametogony functions. At this time, *Et*CDPK4 has physiological and biochemical functions, so parasites generate a large amount of *Et*CDPK4 protein. *Et*CDPK4 protein expression was increased sufficiently to be detected by western blot. Therefore, we propose that although *EtCDPK4* transcripts were moderately expressed in merozoites, *Et*CDPK4 protein could have a high expression in the same stage.

Protein phosphorylation on Ser-/Thr- residues is a key post-translational modification required for signal transduction in eukaryotes [51]. CDPKs have a Ser/Thr kinase domain that phosphorylates downstream substrates. CDPK activity can be detected by either radioactive or non-radioactive methods [52, 53]. In this experiment, the r*Et*CDPK4 activity was detected by non-radioactive methods. r*Et*CDPK4 catalyzed the phosphorylation of the C1-peptide PKC substrate *in vitro* and r*Et*CDPK4 kinase activity could be maintained in a stable range. The kinase activity of r*Et*CDPK4 depended on Ca^2+^ concentration. In the absence of calcium, r*Et*CDPK4 did not have activity. This result was in agreement with results of CDPK kinase activity assays in *Digitaria sanguinalis* mesophyll cells [54]. Therefore, we propose that *Et*CDPK4 activity is completely dependent on calcium in the cytoplasm of *E*. *tenella*.

From the pharmacological perspective, *Et*CDPK4 has unique structural features that could be the target of specific inhibitors in parasites because these targets do not exist in the host. Crystal structures of *Tg*CDPK1 and *Cp*CDPK1 show an enlarged ATP-binding pocket due to glycine at the “gatekeeper” position adjacent to an adenine recognition site [55, 56]. Therefore, the activity of CDPK4 could be blocked by inhibitors. In this study, using a functional module of *Et*CDPK4, we selected seven specific inhibitors: W-7, H-7, H-89, staurosporine, Ro-31-8220, myristoylated peptide and D-sphingosine. The effect of Ca^2+^-concentrations on r*Et*CDPK4 kinase activity and specific inhibitors was determined. The organic compounds W-7, H-7, H-89, staurosporine, Ro-31-8220 and myristoylated peptide significantly decreased enzyme activity, while D-sphingosine had no effect on r*Et*CDPK4 kinase activity. Previous studies reported that activity of *D*. *sanguinalis* CDPKs and the rice CDPK *Os*CDPK14 can be effectively inhibited by W-7 [54, 57] and H-7, staurosporine, Ro-31-8220, and myristoylated peptide inhibit CDPK kinase activity in *D*. *sanguinalis* mesophyll cells [54]. H-89 blocks CDPK kinase activity of French beans in enzyme activity assays *in vitro* [58]. D-sphingosine, a well-known physiological inhibitor of PKC is a broad-spectrum PKC inhibitor. However, the *Et*CDPK4 structure was much more complex than PKC and therefore, the inhibitory effect of D-sphingosine on r*Et*CDPK4 was likely blocked by calcium ions in the reaction system. This might be why D-sphingosine did not inhibit r*Et*CDPK4 activity. This result was similar to findings from previous studies [59] that D-sphingosine does not affect the activity of protein kinase C in porcine theca cells. D-sphingosine did not inhibit enzyme activity but stimulates phospholipase D activity in 7721 human hepatocarcinoma cells [60].

Inhibition of DF-1cell invasion by *E*. *tenella* sporozoites using specific inhibitors of r*Et*CDPK4 showed that W-7, H-7, H-89 and myristoylated peptide significantly decreased sporozoite invasion activity, while staurosporine and Ro-31-8220 had no effect. This result might be because the compounds inhibited the activity of r*Et*CDPK4 *in vitro* but were removed by a regulation mechanism [61] in *E*. *tenella* sporozoites and could not block the kinase activity of *Et*CDPK4 *in vivo*. This would explain the lack of effect on invasion.

We determined experimental conditions for *in vitro* screening of four specific inhibitors r*Et*CDPK4 activity. W-7, H-7, and H-89 are broad-spectrum kinase inhibitors and that affected DF-1 invasion by *E*. *tenella* sporozoites. Myristoylated peptide derived from the pseudosubstrate sequence of β-PKC is a selective inhibitor of PKC subtypes in human fibroblasts [62]. In our study, inhibition by myristoylated peptide was modest but significant for DF-1 invasion by *E*. *tenella* sporozoites. These results showed that the inhibitors effectively blocked the activity of *Et*CDPK4 kinase and inhibited invasion by *E*. *tenella* sporozoite of DF-1 cells.

Expression of *Et*CDPK4 was detected by indirect immunofluorescence assays. Expression of *Et*CDPK4 was high in mature schizonts and forming first-generation merozoites. Therefore, suitable organic compounds that block the *Et*CDPK4 activity may inhibit development of *E*. *tenella* in host cecum epithelial cells. We conclude that *Et*CDPK4 is likely to be an excellent molecular target candidate for anti-coccidiosis drug or vaccine research.

Using antibodies raised against r*Et*CDPK4, we showed by indirect immunofluorescence assays that *Et*CDPK4 kinase was located on the surface and in the cytoplasm of *E*. *tenella* sporozoites and merozoites. CDPKs were found in several subcellular localizations including membranes and cytoplasm and some isoforms were in more than one compartment [40]. Indirect immunofluorescence assays also showed that *Et*CDPK1 [25] and *Et*CDPK3 [24] were near the apical end and on the surface of *E*. *tenella* sporozoites. *Pf*CDPK1 is present in the membrane and organelle fractions of blood-stage parasites and the membrane fraction of ring-stage infected erythrocytes [63, 64]. *Et*CDPK4 has a transmembrane region and three N-myristoylation sites (positions: 8 to 13, 152 to 157, and 253 to 258). Myristoylation sites are vital for membrane targeting and signal transduction in plants and *P*. *falciparum* response to environmental stress or the host cell environment [40, 65]. This might be one reason that *Et*CDPK4 was found on sporozoite and merozoite membranes. *Et*CDPK4 localization in intracellular parasites showed mobilization to the membranes of refractile bodies in the anterior of sporozoites. When *E*. *tenella* developed in DF-1 cells, specific staining was more intense than in trophozoites, mature schizonts or first-generation merozoites, but decreased in immature schizonts by fluorescence intensity. Later during sporozoite development in DF-1 cells, *Et*CDPK4 was found in PV membranes. The PV is a crucial structure that protects the parasite against the host environment [7]. Invasion and the formation of PVs is mediated by different parasite organelles, including micronemes and rhoptries [66]. PV composition evolves during infection according to metabolite exchange between the parasite and host cell [67]. When merozoites escape from mature schizonts to invade new host cells, they must pass through the PV membrane. Thus, we postulated that *Et*CDPK4 was important in merozoite maturity and release. Western blots showed that *Et*CDPK4 protein increased significantly in merozoite stages. This result was similar to localization of *Et*CDPK1 at the apical end of sporozoites after addition to Mardin-Darby bovine kidney cells. *Et*CDPK1 protein was higher in mature schizonts than in immature schizonts. *Et*CDPK1 appeared to be specifically involved in sporozoite invasion of host cells and in release of merozoites from mature *E*. *tenella* schizonts [25].

In *P*. *falciparum*, *Pf*CDPK1 has a key role in schizont development, microneme protein secretion, invasion of host erythrocytes and regulating mRNA expression to assure timely and stage-specific protein expression [68, 69, 70]. *Tg*CDPK7 is crucial for *T*. *gondii* differentiation, growth and proper maintenance of centrosomes [71]. Knock out of *Pb*CDPK3 leads to a pronounced defect in ookinete transmission to the mosquito midgut epithelium and terminates oocysts production [72, 73]. *Pf*CDPK5 is essential for regulating parasite egress from erythrocytes [74], whereas *Pb*CDPK6 is critical for controlling the sporozoite switch from a migratory to an invasion phenotype [75]. Finally, Sharma *et al*. demonstrated that *Pf*CDPK7 binds to PI (4, 5) P2 and controls *P*. *falciparum* development in host erythrocytes [76]. These results suggest that *Et*CDPK4 is related to the invasion and survival of *E*. *tenella* intracellular stages.

Previous *in vitro* invasion inhibition assays showed reduced sporozoite invasion in the presence of monoclonal or specific polyclonal antibodies [34, 36, 77]. In our study, *in vitro* invasion inhibition assays using specific antibody against r*Et*CDPK4 showed partial blockage of the invasion of sporozoites into cells. Inhibition of sporozoites was modest at 52%. Therefore, *Et*CDPK4 might be a key factor in host cell invasion by *E*. *tenella* sporozoites. While further studies are needed to determine the exact function of *Et*CDPK4, because this kinase family is absent from the parasite’s hosts, it represents a target that might be exploited for chemotherapy against Apicomplexans parasites.

In conclusion, we cloned, expressed and characterized a CDPK4 from *E*. *tenella*, adding substantially to the current understanding of its function in the *E*. *tenella* invasion process. Given the importance of *Et*CDPK4 in invasion, host cell adhesion and *E*. *tenella* development in cecum epithelial cells, the results of this study have implications for both novel chemotherapeutic and immunotherapeutic approaches to interfering with *Et*CDPK4 function in *E*. *tenella*.

## References

[1] Elmore SA, Jones JL, Conrad PA, Patton S, Lindsay DS, Dubey JP. *Toxoplasma gondii*: epidemiology, feline clinical aspects, and prevention. Trends Parasitol. 2010; 26: 190–196. 10.1016/j.pt.2010.01.009 20202907

[2] Sachs J, Malaney P. The economic and social burden of *malaria*. Nature. 2002; 415: 680–685. 10.1038/415680a 11832956

[3] Alcala-Canto Y, Ramos-Martinez E, Tapia-Perez G, Gutierrez L, Sumano H. Pharmacodynamic evaluation of a reference and a generic *toltrazuril* preparation in broilers experimentally infected with *Eimeria tenella* or *E*. *acervulina*. Br Poult Sci. 2014; 55: 44–53. 10.1080/00071668.2013.872770 24397403

[4] Matsubayashi M, Hatta T, Miyoshi T, Anisuzzaman, Alim MA, Yamaji K, et al Synchronous development of *Eimeria tenella* in chicken caeca and utility of laser microdissection for purification of single stage schizont RNA. Parasitology. 2012; 139: 1553–1561. 10.1017/S0031182012001072 22906745

[5] Shirley MW, Ivens A, Gruber A, Madeira AM, Wan KL, Dear PH, et al The *Eimeria* genome projects: a sequence of events. Trends Parasitol. 2004; 20: 199–201. 10.1016/j.pt.2004.02.005 15105014

[6] Tabarés E, Ferguson D, Clark J, Soon PE, Wan KL, Tomley F. *Eimeria tenella* sporozoites and merozoites differentially express glycosylphosphatidylinositol-anchored variant surface proteins*1. Mol Biochem Parasitol. 2004; 135:123–132. 15287593 10.1016/j.molbiopara.2004.01.013

[7] Daszak P. Zoite migration during *Eimeria tenella* infection: parasite adaptation to host defences. Parasitol Today. 1999; 15: 67–72. 10234189 10.1016/s0169-4758(98)01379-9

[8] Sasai K, Fetterer RH, Lillehoj H, Matusra S, Constantinoiu CC, Matsubayashi M, et al Characterization of monoclonal antibodies that recognize the *Eimeria tenella* microneme protein MIC2. J Parasitol. 2008; 94: 1432–1434. 10.1645/GE-1558.1 18576850

[9] Moreno SN, Docampo R. Calcium regulation in protozoan parasites. Curr Opin Microbiol. 2003; 6: 359–364. 12941405 10.1016/s1369-5274(03)00091-2

[10] Sanders D, Pelloux J, Brownlee C, Harper JF. Calcium at the crossroads of signaling. Plant Cell. 2002; 14: S401–S417. 10.1105/tpc.002899 12045291 PMC151269

[11] Kudla J, Oliver B, Kenji H. Calcium signals: the lead currency of plant information processing. Plant Cell. 2010; 22: 541–563. 10.1105/tpc.109.072686 20354197 PMC2861448

[12] Hettenhausen C, Sun G, He Y, Zhuang H, Sun T, Qi J, et al Genome-wide identification of calcium-dependent protein kinases in soybean and analyses of their transcriptional responses to insect herbivory and drought stress. Sci Rep. 2016; 6: 18973 10.1038/srep18973 26733237 PMC4702179

[13] Harper JF, Sussman MR, Schaller GE, Putnam-Evans C, Charbonneau H, Harmon AC. A calcium-dependent protein kinase with regulatory domain similar to calmodium. Science. 1991; 252: 951–954. 1852075 10.1126/science.1852075

[14] Cheng SH, Willmann MR, Chen HC, Sheen J. Calcium signaling through protein kinases. The *Arabidopsis* calcium-dependent protein kinase gene family. Plant Physiol. 2002; 129: 469–485. 10.1104/pp.005645 12068094 PMC1540234

[15] Harper JF, Huang JF, Lloyd SJ. Genetic identification of an autoinhibitor in CDPK, a protein kinase with a calmodulin-like domain. Biochemistry. 1994; 33:7267–7277. 8003490 10.1021/bi00189a031

[16] Lourido S, Shuman J, Zhang C, Shokat KM, Hui R, Sibley LD. Calcium-dependent protein kinase 1 is an essential regulator of exocytosis in *Toxoplasma*. Nature. 2010; 465: 359–362. 10.1038/nature09022 20485436 PMC2874977

[17] Lourido S, Tang K, Sibley LD. Distinct signalling pathways control *Toxoplasma* egress and host-cell invasion. EMBO J. 2012; 31: 4524–4534. 10.1038/emboj.2012.299 23149386 PMC3545288

[18] Billker O, Dechamps S, Tewari R, Wenig G, Franke-Fayard B. Calcium and a calcium-dependent protein kinase regulate gamete formation and mosquito transmission in a *malaria* parasite. Cell. 2004; 117: 503–514. 15137943 10.1016/s0092-8674(04)00449-0

[19] Mccoy JM, Whitehead L, van Dooren GG, Tonkin CJ. Correction: *Tg*CDPK3 Regulates Calcium-Dependent Egress of *Toxoplasma gondii* from Host Cells. PLoS Pathog. 2013; 8: 155–170. 10.1371/journal.ppat.1003066 PMC3514314 23226109

[20] Erin G, Moritz T, Emma E, Butz H, Garbuz T, Oswald BP, et al A Forward Genetic Screen Reveals that Calcium-dependent Protein Kinase 3 Regulates Egress in *Toxoplasma*. PLoS Pathog. 2012; 8: e1003049–e1003049 10.1371/journal.ppat.1003049 23209419 PMC3510250

[21] Moritz T, Sanders JL, Gaji RY, Lafavers KA, Child MA. The calcium-dependent protein kinase 3 of *Toxoplasma* influences basal calcium levels and functions beyond egress as revealed by quantitative phosphoproteome analysis. PLoS Pathog. 2014; 10: e1004197–e1004197. 10.1371/journal.ppat.1004197 24945436 PMC4063958

[22] Nagamune K, Sibley LD. Comparative genomic and phylogenetic analyses of calcium ATPases and calcium-regulated proteins in the Apicomplexa. Mol Biol Evol. 2006; 23: 1613–1627. 10.1093/molbev/msl026 16751258

[23] Billker O, Lourido S, Sibley LD. Calcium-dependent signaling and kinases in Apicomplexan parasites. Cell Host Microbe. 2009; 5: 612–622. 10.1016/j.chom.2009.05.017 19527888 PMC2718762

[24] Han HY, Zhu SH, Jiang LL, Li Y, Dong H, Zhao QP, et al Molecular characterization and analysis of a novel calcium-dependent protein kinase from *Eimeria tenella*. Parasitology. 2013; 140: 746–755. 10.1017/S0031182012002107 23369433

[25] Dunn PPJ, Bumstead JM, Tomley FM. Sequence, expression and localization of calmodulin-domain protein kinases in *Eimeria tenella* and *Eimeria maxima*. Parasitology. 1996; 113: 439–448. 8893529 10.1017/s0031182000081506

[26] Harper JF, Harmon A. Plants, symbiosis and parasites: a calcium signalling connection. Nat Rev Mol Cell Biol. 2005; 6: 555–566. 10.1038/nrm1679 16072038

[27] Zhang Z, Ojo KK, Vidadala R, Huang W, Geiger JA, Scheele S, et al Potent and selective inhibitors of CDPK1 from *T*. *gondii* and *C*. *parvum* based on a *5-aminopyrazole-4-carboxamide* scaffold. ACS Med Chem Lett. 2014; 5:40–44. 10.1021/ml400315s 24494061 PMC3908674

[28] Hui R, Bakkouri ME, Sibley LD. Designing selective inhibitors for calcium-dependent protein kinases in apicomplexans. Trends Pharmacol Sci. 2015; 36: 452–460. 10.1016/j.tips.2015.04.011 26002073 PMC4485940

[29] Tomley F. Techniques for isolation and characterization of apical organelles from *Eimeria tenella* sporozoites. Methods. 1997; 13: 171–176. 10.1006/meth.1997.0509 9405200

[30] Xie MQ, Gilbert JM, Fuller AL, McDougald LR. A new method for purification of *Eimeria tenella* merozoites. Parasitol Res. 1990; 76: 566–569. 2217116 10.1007/BF00932562

[31] Jiang L, Lin J, Han H, Zhao Q, Dong H, Zhu S, et al Identification and partial characterization of a serine protease inhibitor (serpin) of *Eimeria tenella*. Parasitol Res. 2012; 110: 865–874. 10.1007/s00436-011-2568-0 21842392

[32] Ranjan R, Ahmed A, Gourinath S, Sharma P. Dissection of mechanisms involved in the regulation of *Plasmodium falciparum* calcium-dependent protein kinase 4. J Biol Chem. 2009; 284: 15267–15276. 10.1074/jbc.M900656200 19307175 PMC2685707

[33] Jiang LL, Lin JJ, Han HY, Dong H, Zhao QP, Zhu SH, et al Establishment and application of DF-1 cell culture system for the sporozoites of *Eimeria tenella*. Vet Sci China. 2011; 41: 551–556.

[34] Jahn D, Matros A, Bakulina AY, Tiedemann J, Schubert U, Giersberg M, et al Model structure of the immunodominant surface antigen of *Eimeria tenella* identified as a target for sporozoite-neutralizing monoclonal antibody. Parasitol Res. 2009; 105: 655–668. 10.1007/s00436-009-1437-6 19387686

[35] Labbé M, Péroval M, Bourdieu C, Girard-Misguich F, Péry P. *Eimeria tenella* enolase and pyruvate kinase: A likely role in glycolysis and in others functions. Int J Parasitol. 2006; 36: 1443–1452. 10.1016/j.ijpara.2006.08.011 17030033

[36] Péroval M, Péry P, Labbé M. The heat shock protein 90 of *Eimeria tenella* is essential for invasion of host cell and schizont growth. Int J Parasitoly. 2006; 36: 1205–1215. 10.1016/j.ijpara.2006.04.006 16753167

[37] Etzold M, Lendner M, Daugschies A, Dyachenko V. CDPKs of *Cryptosporidium parvum*—stage-specific expression *in vitro*. Parasitol Res. 2014; 113: 2525–2533. 10.1007/s00436-014-3902-0 24810092

[38] Dammann C, Ichida A, Hong B, Romanowsky SM, Hrabak EM. Subcellular targeting of nine calcium-dependent protein kinase isoforms from *Arabidopsis*. Plant Physiol. 2003; 132: 1840–1848. 10.1104/pp.103.020008 12913141 PMC181270

[39] Hrabak EM, Dickmann LJ, Satterlee JS, Sussman MR. Characterization of eight new members of the calmodulin-like domain protein kinase gene family from *Arabidopsis thaliana*. Plant Mol Biol. 1996; 31: 405–412. 8756605 10.1007/BF00021802

[40] Möskes C, Burghaus PA, Wernli B, Sauder U, Dürrenberger M, Kappes B. Export of *Plasmodium falciparum* calcium-dependent protein kinase 1 to the parasitophorous vacuole is dependent on three N-terminal membrane anchor motifs. Mol Microbiol. 2004; 54: 676–691. 10.1111/j.1365-2958.2004.04313.x 15491359

[41] De Sousa Abreu R, Penalva LO, Marcotte EM, Vogel C. Global signatures of protein and mRNA expression levels. Mol Biosyst. 2009; 5: 1512–1526. 10.1039/b908315d 20023718 PMC4089977

[42] Li JL, Baker DA, Cox LS. Sexual stage-specific expression of a third calcium-dependent protein kinase from *Plasmodium falciparum*. Biochim Biophys Acta. 2000; 1491: 341–349. 10760601 10.1016/s0167-4781(00)00032-4

[43] Kieschnick H, Wakefield T, Narducci CA, Beckers C. *Toxoplasma gondii* attachment to host cells is regulated by a calmodulin-like domain protein kinase. J Biol Chem. 2001; 276: 12369–12377. 10.1074/jbc.M011045200 11154702

[44] Donald RG, Zhong T, Wiersma H, Nare B, Yao D, Lee A, et al Anticoccidial kinase inhibitors: identification of protein kinase targets secondary to cGMP-dependent protein kinase. Mol Biochem Parasitol. 149: 86–98. 10.1016/j.molbiopara.2006.05.003 16765465

[45] Wilson PAG, Fairbairnz D. Biochemistry of Sporulation in Oocysts of *Eimeria acervulina**. J Eukaryot Microbiol. 2007; 8: 410–416.

[46] Belli SI, Walker RA, Flowers SA. Global protein expression analysis in apicomplexan parasites: current status. Proteomics. 2005; 5: 918–924. 10.1002/pmic.200401161 15759314

[47] Michalski WP, Edgar JA, Prowse SJ. Mannitol metabolism in *Eimeria tenella*. Int J Parasitol. 1992; 22: 1157–1163. 1487375 10.1016/0020-7519(92)90035-j

[48] Mielke D, Alabdul RG. The sporogony of *Eimeria tenella*. Angew Parasitol. 1991; 32: 39–41. 2039092

[49] Katrib M, Ikin RJ, Brossier F, Robinson M, Slapetova I, Sharman PA, et al Stage-specific expression of protease genes in the apicomplexan parasite, *Eimeria tenella*. BMC genomics. 2012; 13: 685 10.1186/1471-2164-13-685 23216867 PMC3770453

[50] Fetterer RH, Miska KB, Lillehoj H, Barfield RC. Serine protease activity in developmental stages of *Eimeria tenella*. J Parasitol. 2007; 93: 333–340. 10.1645/GE-824R1.1 17539417

[51] Nemoto K, Takemori N, Seki M, Shinozaki K, Sawasaki T. Members of the Plant CRK-superfamily are Capable of trans-/auto-Phosphorylation of Tyrosine Residues. J Biol Chem. 2015; 290: 16665–16677. 10.1074/jbc.M114.617274 25969537 PMC4505418

[52] Keyloun KR, Reid MC, Choi R, Song Y, Fox AM, Hillesland HK, et al The gatekeeper residue and beyond: homologous calcium-dependent protein kinases as drug development targets for veterinarian Apicomplexa parasites. Parasitology. 2014; 141: 1499–1509. 10.1017/S0031182014000857 24927073 PMC4390295

[53] Leclercq J, Ranty B, Sanchez-Ballesta MT, Li Z, Jones B, Jauneau A, et al Molecular and biochemical characterization of *Le*CRK1, a ripening-associated tomato CDPK-related kinase. J Exp Bot. 2005; 56: 25–35. 10.1093/jxb/eri003 15501910

[54] Osuna L, Coursol S, Pierre JN, Vidal J. A Ca^2+^-dependent protein kinase with characteristics of protein kinase C in leaves and mesophyll cell protoplasts from *Digitaria sanguinalis*: possible involvement in the C4-phosphoenolpyruvate carboxylase phosphorylation cascade. Biochem Bioph Res Co. 2004; 314: 428–433. 10.1016/j.bbrc.2003.12.103 14733923

[55] Ojo KK, Larson ET, Keyloun KR, Castaneda LJ, Derocher AE, Inampudi KK, et al *Toxoplasma gondii* calcium-dependent protein kinase 1 is a target for selective kinase inhibitors. Nat Struct Mol Biol. 2010; 17: 602–607. 10.1038/nsmb.1818 20436472 PMC2896873

[56] Wernimont AK, Artz JD, Finerty P Jr, Lin YH, Amani M, Allali-Hassani A, et al Structures of Apicomplexan calcium-dependent protein kinases reveal mechanism of activation by calcium. Nat Struct Mol Biol. 2010; 17: 596–601. 10.1038/nsmb.1795 20436473 PMC3675764

[57] Zhang T, Wang Q, Chen X, Tian C, Wang X, Xing T, et al Cloning and biochemical properties of CDPK gene *Os*CDPK14 from *rice*. J Plant Physiol. 2005; 162: 1149–1159. 10.1016/j.jplph.2004.12.010 16255173

[58] Allwood EG, Davies DR, Gerrish C, Bolwell GP. Regulation of CDPKs, including identification of PAL kinase, in biotically stressed cells of *French bean*. Plant Mol Biol. 2002; 49: 533–544. 12090628 10.1023/a:1015502117870

[59] Kaminski T. The response of phospholipase C/protein kinase C and adenylyl cyclase/protein kinase A pathways in porcine theca interna cells to opioid agonist FK 33–824. Domest Anim Endocrinol. 2004; 27: 379–396. 10.1016/j.domaniend.2004.05.001 15519041

[60] Huang Y, Zhang X, Chen H. Regulation of phospholipase D activity in *human* hepatocacinoma cells by protein kinases and *D-sphingosine*. Acta Bioch Bioph Sin. 1998; 31: 572–576. 12114973

[61] Salowe SP, Judyann W, Liberator PA, Donald RGK. The role of a parasite-specific allosteric site in the distinctive activation behavior of *Eimeria tenella* cGMP-dependent protein kinase. Biochemistry. 2002; 41: 4385–4391. 11914085 10.1021/bi0156658

[62] Eichholtz T, de Bont DB, de Widt J, Liskamp RM, Ploegh HL. A myristoylated pseudosubstrate peptide, a novel protein kinase C inhibitor. J Biol Chem. 1993; 268: 1982–1986. 8420972

[63] Yi Z, Franklin RM, Kappes B. *Plasmodium falciparum* calcium-dependent protein kinase phosphorylates proteins of the host erythrocytic membrane. Mol Biochem Parasitol. 1994; 66: 329–343. 7808482 10.1016/0166-6851(94)90159-7

[64] Zhao Y, Pokutta S, Maurer P, Lindt M, Franklin RM, Kappes B. Calcium-binding properties of a calcium-dependent protein kinase from *Plasmodium falciparum* and the significance of individual calcium-binding sites for kinase activation. Biochemistry. 1994; 33: 3714–3721. 8142371 10.1021/bi00178a031

[65] Podell S, Gribskov M. Predicting N-terminal myristoylation sites in plant proteins. BMC Genomics. 2004; 5: 1–15. 15202951 10.1186/1471-2164-5-37 PMC449705

[66] Carruthers VB, Sibley LD. Sequential protein secretion from three distinct organelles of *Toxoplasma gondii* accompanies invasion of *human* fibroblasts. Eur J Cell Biol. 1997; 73:114–23. 9208224 PMC13475723

[67] Entzeroth R, Mattig FR, Wernermeier R. Structure and function of the parasitophorous vacuole in *Eimeria species*. Int J Parasitol. 1998; 28: 1015–1018. 9724871 10.1016/s0020-7519(98)00079-4

[68] Abhisheka B, Shailja S, More KR, Dhiraj H, Kuldeep N. Characterization of *Plasmodium falciparum* calcium-dependent protein kinase 1 (*Pf*CDPK1) and its role in microneme secretion during erythrocyte invasion. J Biol Chem. 2013; 288: 1590–1602. 10.1074/jbc.M112.411934 23204525 PMC3548469

[69] Sebastian S, Brochet M, Collins M, Schwach F, Jones ML, Goulding D, et al A *Plasmodium* Calcium-Dependent Protein Kinase Controls Zygote Development and Transmission by Translationally Activating Repressed mRNAs. Cell Host Microbe. 2012; 12: 9–19. 10.1016/j.chom.2012.05.014 22817984 PMC3414820

[70] Azevedo MF, Sanders PR, Efrosinia K, Nie CQ, Fu P, Bach LA, et al Inhibition of *Plasmodium falciparum* CDPK1 by conditional expression of its J-domain demonstrates a key role in schizont development. Biochem J. 2013; 452: 433–441. 10.1042/BJ20130124 23548171

[71] Juliette M, Laurence B, Chen C, Gubbels MJ, Lebrun M, Daher W. The *Toxoplasma gondii* calcium-dependent protein kinase 7 is involved in early steps of parasite division and is crucial for parasite survival. Cell Microbiol. 2014; 16: 95–114. 10.1111/cmi.12186 24011186 PMC4091637

[72] Ishino T, Orito Y, Chinzei Y, Yuda M. A calcium-dependent protein kinase regulates *Plasmodium* ookinete access to the midgut epithelial cell. Mol Microbiol. 2006; 59: 1175–1184. 10.1111/j.1365-2958.2005.05014.x 16430692

[73] Siden-Kiamos I, Ecker A, Nybäck S, Louis C, Sinden RE, Billker O. *Plasmodium berghei* calcium-dependent protein kinase 3 is required for ookinete gliding motility and mosquito midgut invasion. Mol Microbiol. 2006; 60: 1355–1363 10.1111/j.1365-2958.2006.05189.x 16796674 PMC1513514

[74] Dvorin JD, Martyn DC, Patel SD, Grimley JS, Collins CR, Hopp CS, et al A plant-like kinase in *Plasmodium falciparum* regulates parasite egress from erythrocytes. Science. 2010; 328: 910–912. 10.1126/science.1188191 20466936 PMC3109083

[75] Coppi A, Tewari R, Bishop JR, Bennett BL, Lawrence R, Esko JD, et al Heparan sulfate proteoglycans provide a signal to *Plasmodium* sporozoites to stop migrating and productively invade host cells. Cell Host Microbe. 2007; 2: 316–327. 10.1016/j.chom.2007.10.002 18005753 PMC2117360

[76] Kumar P, Tripathi A, Ranjan R, Halbert J, Gilberger T, Doerig C, et al Regulation of *Plasmodium falciparum* development by calcium-dependent protein kinase 7 (*Pf*CDPK7). J Biol Chem. 2014; 289: 20386–20395. 10.1074/jbc.M114.561670 24895132 PMC4106351

[77] Jiang L, Lin J, Han H, Dong H, Zhao Q, Zhu S, et al Identification and Characterization of *Eimeria tenella* Apical Membrane Antigen-1 (AMA1). PLoS One. 2012; 7:362–364. 10.1371/journal.pone.0041115 PMC3400601 22829917

